# Outcomes of Geriatric Patients with Hepatocellular Carcinoma

**DOI:** 10.3390/curroncol29060346

**Published:** 2022-06-16

**Authors:** Chern-Horng Lee, Tzung-Hai Yen, Sen-Yung Hsieh

**Affiliations:** 1 Division of General Internal Medicine and Geriatrics, Chang Gung Memorial Hospital, Linkou Branch, Taoyuan 333, Taiwan; lee4570@cgmh.org.tw; 2 Department of Nephrology, Clinical Poison Center, Chang Gung Memorial Hospital, Linkou Branch, Taoyuan 333, Taiwan; 3 College of Medicine, Chang Gung University, Taoyuan 333, Taiwan; 4 Department of Gastroenterology and Hepatology, Chang Gung Memorial Hospital, Linkou Branch, Taoyuan 333, Taiwan

**Keywords:** hepatocellular carcinoma, geriatric patients, mortality

## Abstract

Background: The treatment modalities and outcomes of geriatric patients with hepatocellular carcinoma (HCC) remain controversial. This retrospective observational cohort study compared the outcomes of HCC between geriatric and younger patients. Methods: The medical records of patients with HCC managed between January 2001 and December 2017 were retrieved from the Chang Gung Memorial Hospital Research Database. Patients were stratified by age into two groups: a geriatric group (65–75 years) and a younger group (<65 years). The two groups were matched through 1:2 propensity score matching (PSM) according to sex, cardiovascular disease, cerebrovascular attack, diabetes mellitus, cirrhosis, hepatitis, and hypertension. Results: Of the 11,033 patients with HCC, 2147 patients aged 65–75 years and 4294 patients aged <65 years were identified after 1:2 PSM. The Kaplan–Meier model revealed that the HCC outcomes in patients older than 65 years were not significantly different after 3 years (*p* = 0.060). Consistent results were also obtained when the laboratory data associated with HCC incidence were included in the Fine–Gray competing risk model after 1:2 PSM (*p* = 0.1695). The major risk factors for HCC survival were systemic immune-inflammation index (SII) ≥ 610 × 10^9^ cells/L, advanced tumor stage, and model for end-stage liver disease (MELD) score, etc. Conclusion: Age was not an independent factor for mortality in patients with HCC in the first 3 years. Geriatric patients with HCC should be as aggressively managed as younger patients.

## 1. Introduction

The incidence of cancer is elevated 11-fold in adults older than 65 years compared with those 65 years or younger [1]. In hepatocellular carcinoma (HCC), the incidence increases with age [2], and the risk of HCC increases more than 15-fold after 65 years in patients infected with hepatitis C [3]. The global median survival and 1-, 3-, and 5-year survival rates of older HCC patients were 27 months and 71%, 36%, and 16%, respectively, which were worse than those of younger patients (33 months, 77%, 44%, and 21%, respectively) (*p* = 0.002) [4].

The HCC patients with BCLC stage 0 and stage A disease generally underwent operation, hepatic transplantation, or interventional treatments for local tumor, comprising radiofrequency ablation, ethanol or acetic acid injection, and transcatheter arterial chemo-embolization [5,6]. Patients with BCLC stage B disease undertook transcatheter arterial chemo-embolization and radiofrequency ablation. Patients with BCLC stage C disease received palliative chemotherapy, transcatheter arterial chemo-embolization, or radiotherapy along with supportive medications, and those with BCLC stage D disease obtained palliative medications. A new therapeutic option for unresectable HCC is immunotherapy. HCC is a classic example of inflammation-linked malignancy, and the tumor microenvironment is infiltrated with diverse kinds of immune active cells, for example, T cells, natural killer cells, myeloid cells, etc. [7]. Currently, there are some published or ongoing clinical trials evaluating the benefit of dual immune checkpoint blockade or a combination of immune checkpoint inhibitors and biological therapy in patients with unresectable HCC [8,9]. Furthermore, De Lorenzo et al. reported that metronomic capecitabine therapy could be another option for HCC patients with Child–Pugh B liver cirrhosis [10].

It has been suggested that a geriatric assessment influences oncological treatment decisions, limiting treatment intensity in vulnerable patients as well as preventing under-treatment of fit patients. Geriatric patients may also present with comorbidities, and their condition may deteriorate owing to cancer-unrelated causes [1]. Missed or delayed diagnosis of malignancy can occur in geriatric patients who are not adequately managed. Older age, high Eastern Cooperative Oncology Group (ECOG) score, and advanced Barcelona Clinic Liver Cancer classification (BCLC) stage were associated with poorer prognosis in a cohort study [4]. However, Guo et al. [4] reported that overall survival was not significantly different between older and younger patients within similar BCLC stages or after similar treatments. Survival differences between geriatric and younger patients have hence remained controversial. This study investigated the outcomes of HCC in geriatric patients. To evaluate the outcomes of geriatric patients with HCC, we used 1:2 propensity score matching (PSM) to divide patients randomly into two groups: a geriatric group (65–75 years) and a younger group (<65 years).

## 2. Materials and Methods

### 2.1. Ethics Statement

All patient-identifiable information was encrypted by de-linking it from the main dataset, and the information was available only to the investigators. HCC cases were retrieved using ICD-9 (from 1997 to 2015) and ICD-10 (since 2016) codes (listed in the Table S1), and the diagnoses were required to have been diagnosed at least five times in outpatient clinics or once during hospitalization. The drugs were identified according to WHO anatomical therapeutic chemical codes. The Chang Gung Research Database (CGRD) was linked to the databases of the National Patient Registry, Cancer Registry, and Prescribed Drug Registry. The Institutional Review Board of the Chang Gung Medical Foundation approved the study and waived the requirement for written informed consent.

### 2.2. Study Population

Data from 1 January 2003 to 31 December 2017 were retrieved from the CGRD. The CGRD, a regularly updated and well-validated tool, contains comprehensive diagnostic data, laboratory test results, prescription history, and both outpatient and hospitalization information of patients with long-term follow up at six major hospitals in different regions of Taiwan. The inclusion criteria were clinical and/or pathological diagnosis of HCC and complete medical records, which was determined on the basis of the inclusion of all of the following information: patient age and sex; alpha-fetoprotein (AFP) level; complete blood count (CBC); albumin level; bilirubin level; prothrombin time (PT); creatinine (Cr) level; aspartate aminotransferase (AST) level; alanine aminotransferase (ALT) level; serum hepatitis B virus surface antigen (HBsAg); antibodies to the hepatitis C virus (anti-HCV); tumor, node, and metastasis (TNM) staging; number of tumors; largest tumor size; presence of liver cirrhosis; and date of last follow-up or HCC-related death. For the systemic immune-inflammation index (SII) (neutrophil × platelet/lymphocyte), we selected 610 × 10^9^/L as the cut-off value in the receiver operating characteristic curve analysis [11].

### 2.3. Tumor Staging System

We stratified the HCC stages according to the tumor (T), nodes (N), and metastases (M) staging system because the system was used in the CGRD.

### 2.4. Inclusion Criteria

**Figure 1:**
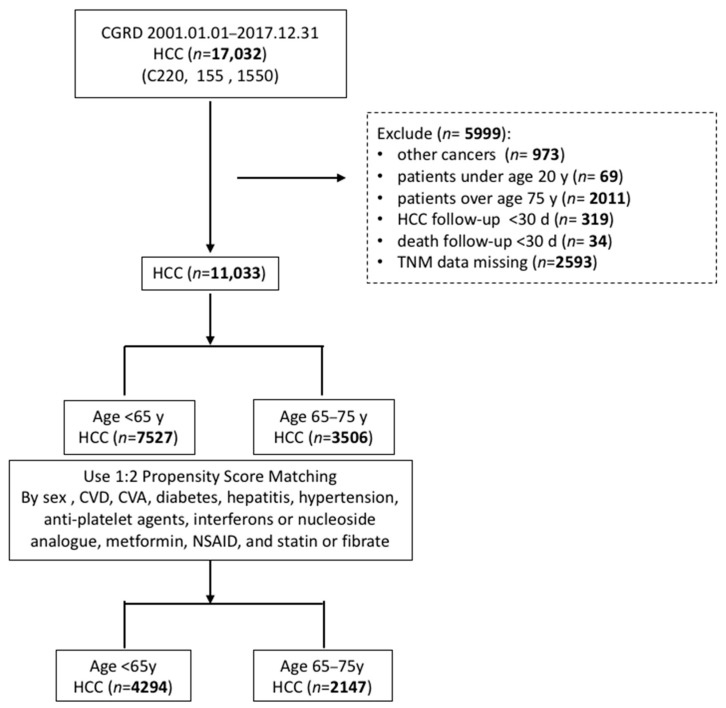
Flowchart of the study population.

HCC (*n* = 17,032) retrieved using ICD-9 (155) and ICD-10 (C220) codes (listed in the Table S1) were required to have been diagnosed at least five times in outpatient clinics or once during hospitalization (Figure 1). The drug use duration was at least 84 days in our study according to the reimbursement policy of the National Health Insurance Program, Taiwan. Drug therapy was defined when a patient had more than three outpatient claims. Comorbidities were required to have been diagnosed at least three times in outpatient clinics or once during hospitalization. A total of 7527 patients (<65 years) and 3506 patients (65–75 years) were randomly matched through propensity score matching (1:2) according to sex, cardiovascular disease, cerebrovascular attack, diabetes mellitus, and treatment with metformin or statins. We further added laboratory data related to HCC occurrence, including serum ALT (hepatitis activity), platelet count (liver fibrosis degree), total bilirubin, and prothrombin time (cirrhosis severity), to the matching score. Thus, 4294 patients were included in the younger group, and 2147 patients were included in the geriatric group (laboratory data were not included for PSM).

### 2.5. Exclusion Criteria

A total of 5999 patients were excluded according to the following criteria: other cancers, 973; age < 20 years, 69; age > 75 years, 2011; HCC follow-up < 30 days, 319; death during follow-up < 30 days, 34; and missing TNM stage, 2593 (Figure 1).

### 2.6. Statistical Analysis

We used statistical software R version 2.15.1 for the initial analyses. Univariate and multivariate Cox regression analyses were used to examine independent risk factors for predicting HCC outcomes. All analyses were performed using SAS statistical package version 9.4 (SAS Institute Inc., Cary, NC, USA). The significance level was set at a 2-tailed *p*-value of <0.05.

## 3. Results

### 3.1. Patient Selection

We enrolled CGRD cohorts of patients with HCC from six hospital branches in different regions of Taiwan. After the exclusion criteria were applied, 11,033 eligible patients were included in the subsequent analyses (Figure 1). We classified the patients into two age groups, and their basic characteristics are presented in Table 1. A PSM analysis was conducted according to sex, cardiovascular disease, cerebrovascular attack, diabetes mellitus, cirrhosis, hepatitis, hypertension, anti-platelet agent, interferon or nucleic analog used, metformin, non-steroidal anti-inflammatory drug (NSAID), and statin or fibrate (Table 1).

### 3.2. Outcome

**Figure 2:**
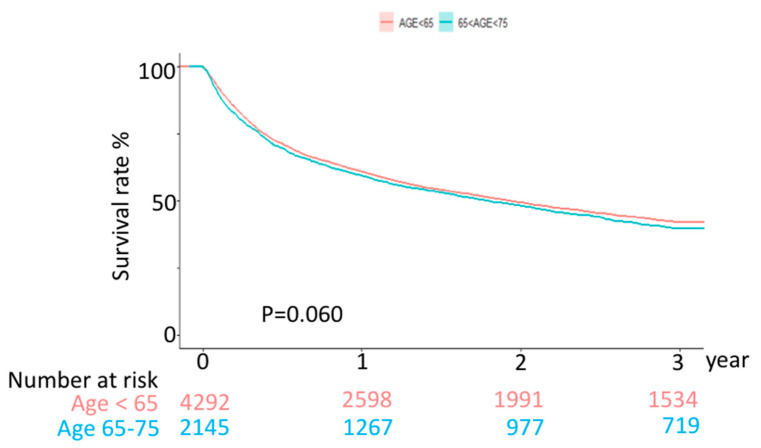
Comparison of overall HCC mortality incidences between geriatric and younger patients after 3-year follow-up.

The survival rate was not different between geriatric and younger patients after 3 years, *p* = 0.1695, based on the Fine–Gray competing risk model after 1:2 PSM (Table 2) and not significant, *p* = 0.060, based on the Kaplan–Meier model (Figure 2).

### 3.3. Risk Factors by Multivariate Cox Regression Analysis

The initial univariate analysis revealed different significant risk factors associated with survival between both groups. Therefore, we entered these significant risk factors into a multivariate Cox regression analysis, which revealed significantly increased risk factors for HCC mortality after 3 years, namely sex (HR: 1.5380, *p* < 0.0001), cirrhosis (HR: 1.5950, *p* < 0.0001), AFP (HR: 1.6460, *p* < 0.0001), AST (HR: 1.7870, *p* < 0.0001), total bilirubin (HR: 1.8520, *p* < 0.0001), INR > 1.7 (HR: 2.3280, *p* < 0.0001), SII > 610 × 10^9^ cells/L (HR: 1.5180, *p* < 0.0001), TNM stage (II, HR: 1.7430, *p* < 0.0001; III, HR: 5.4860, *p* < 0.0001; IV, HR: 9.6060, *p* < 0.0001), and MELD score (2 points, HR: 1.3970, *p* = 0.0079; 3 points, HR: 1.6780, *p* = 0.0002) (Table 3). Significantly decreased risk factors for HCC mortality were as follows: interferon or nucleoside analog used (HR: 0.7620, *p* = 0.0016), statin or fibrate used (HR: 0.5710, *p* = 0.0029), and albumin (HR: 0.7410, *p* = 0.0001).

## 4. Discussion

Geriatric patients were traditionally thought to have worse HCC outcomes owing to increased comorbidities, including cardiovascular disease, pulmonary disease, diabetes mellitus, and renal insufficiency [12]. Older patients are more likely to be strictly selected for aggressive treatments probably because of poor general condition, preexisting comorbidities, increased toxicity during chemotherapy [13], reluctance of clinicians/patients to offer/undergo surgery [4], longer hospital stay, and higher in-hospital mortality rates [14]. Identifiable comorbidities and frailty changes due to aging can limit a patient’s ability to tolerate cancer therapy [15].

The analytical result is important because it supports similar survival outcomes between geriatric and younger patients with HCC in the first 3 years. In a retrospective study of 1530 patients with HCC, Guo et al. [4] stated that geriatric patients showed comparable survival to younger patients following BCLC stage or treatment stratification. The performance status, BCLC stage, and treatment but not age were significant predictors for survival in the cohort patients. Compared with patients younger than 70 years, older patients demonstrated similar 3-year survival after resection and ablation [12]. The Geriatric Index of Comorbidity also predicted mortality (relative risk of death: 2.3, 95% confidence interval (CI): 1.7–3.1) [16]; hence, careful management must be conducted according to individual geriatric conditions. The five-year graft survival rates after adjustment for death as a competing risk for ages 50–54 years, 55–59 years, 60–64 years, 65–69 years, and >70 years were 85.8%, 87.3%, 89.6%, 89.1%, and 88.9%, respectively [17].

In a study of geriatric patients with solitary HCC who underwent surgery, Zarour et al. [18] found that the median survival was 72 months and that the 3-year survival was 59%. Furthermore, it was found that a higher blood AFP level and a lower Prognostic Nutritional Index, which is based on the serum albumin concentration and peripheral blood lymphocyte count, were linked with overall survival. In this study, the survival rates of geriatric patients were 40.89% and 20.07% after 3 and 5 years, respectively, compared with those of younger patients, which were 43.19% and 26.24% after 3 and 5 years, respectively.

After PSM, we observed that geriatric patients with HCC did not have worse outcomes than younger patients in the first 3 years. In a study of 439 HCC patients who underwent surgery, Famularo et al. [19] disclosed no significant differences in overall, tumor-specific, and recurrence-free survival between geriatric and younger patients. Aging was the variable correlated with post-surgical complications, and hepatic-related morbidity was a significant determinant of overall survival. Therefore, it was suggested that aging is not a contraindication for hepatic operation per se but necessitates careful patient selection before surgery. In another study of 450 HCC patients, Seo et al. [20] revealed that the performance score, MELD score, modified Union for International Cancer Control staging, BCLC staging, presence of portal vein tumor thrombosis, ruptured HCC, and treatment were predictors of overall survival. Therefore, it was suggested that standard treatment should be offered to any HCC patient irrespective of age.

We identified male sex, cirrhosis, AFP ≥ 100 µg/L, AST ≥ 70 u/dL, total bilirubin (>2.0 mg/dL), INR > 1.7, SII > 610 × 10^9^ cells/L, advanced tumor stage TNM, and MELD score as risk factors for poor outcomes. However, significantly decreased risk factors for HCC mortality, such as interferon or nucleic analog used, statin or fibrate used, and albumin (>3.0 g/L), indicated better outcomes. The overall survival in the first 3 years was similar between geriatric and younger patients; thus, survival might depend on performance status, liver function, TNM tumor stages, and effective treatments rather than age, as demonstrated by these independent factors determining prognosis in the cohort study [4,21].

According to a review paper [22], Desai and Lichtman confirmed that geriatric patients with HCC could be as safely treated with standard therapy as in the younger patients. A higher SII was significantly associated with vascular invasion, large tumors, and early recurrence [11]. Patients with HCC who had SII > 610 × 10^9^ cells/L had a shorter survival time than those with SII < 610 × 10^9^ cells/L.

HCC exhibits a high recurrence or metastasis rate of more than 50% within the first 3 years, with recurrence rates of approximately 70% and 74% at 3 and 5 years after primary curative therapy, respectively [11,23]. Our results showed that geriatric patients with HCC had no more significant outcomes than young patients after 3 years; hence, all patients with HCC must be aggressively managed regardless of age.

The data in the literature on the survival difference between geriatric and younger patients remains controversial. Nevertheless, this retrospective observational cohort study has confirmed that age was not an independent factor for mortality in patients with HCC in the first 3 years. The major risk factors for HCC survival were SII ≥ 610 × 10^9^ cells/L, advanced tumor stage, and MELD score, etc. Therefore, it is suggested that geriatric patients with HCC should be as aggressively managed as the younger patients. Apart from standard conventional anti-cancer therapy, immunotherapy is a new therapeutic option for unresectable HCC. As mentioned, comorbidities and frailty changes due to aging can limit a patient’s ability to endure conventional therapy, such as surgery or systemic chemotherapy. Therefore, immunotherapy could be a promising treatment for geriatric patients in future.

The limitations of this study include the retrospective study design; the small sample size; and the lack of data on the BCLC stage, the Child–Pugh score, and the treatment modalities. Large-scale prospective studies are necessary to confirm this observation.

## 5. Conclusions

Although age was not an independent factor for HCC outcomes in the first 3 years, geriatric HCC should still be managed aggressively, and diagnosis must not be delayed. The treatment objectives for geriatric patients with HCC should be set according to liver reserve function, comorbidity, and tumor characteristics. Increasing the geriatric cutoff age to 68 years may be considered, pending further evidence.

## Figures and Tables

**Table 1:** Baseline HCC characteristics between geriatric and younger patients after matching (1:2), *N* = 6441.

Age	<65 Years (*N* = 4294)		65–75 Years (*N* = 2147)		*p*-Value
**Sex (*n*, %)**					0.7651
Male	3521	54.67%	1767	27.43%	
Female	773	12.00%	380	5.90%	
**TNM stage (*n*, %)**					0.8804
I	1395	21.66%	697	10.82%	
II	851	13.21%	444	6.89%	
III	1272	19.75%	625	9.70%	
IV	776	12.05%	381	5.92%	
**Comorbidities (*n*, %)**					
CAD or CVA	53	0.82%	40	0.62%	0.0461
Diabetes	751	11.66%	373	5.79%	0.9076
Cirrhosis	2787	43.27%	1404	21.80%	0.698
Hepatitis	257	3.99%	126	1.96%	0.8522
Hypertension	648	10.06%	321	4.98%	0.8824
**Medications use (*n*, %)**					
Anti-platelet or Aspirin	595	9.24%	262	4.07%	0.0655
Interferons or nucleoside analogue	1110	17.23%	301	4.67%	<0.0001
Metformin	413	6.41%	198	3.07%	0.6092
NSAID	2904	45.09%	1282	19.90%	<0.0001
Statin or fibrate	161	2.50%	63	0.98%	0.0924
**Laboratory data (median, Q1 to Q3)**					
AFP (ng/mL) ≥ 100	34.16	7.24–535.5	28.15	6.13–304.2	0.0054
ALT (U/L) ≥ 70	47.00	30–76	45.00	29–81	0.5408
AST (U/L) ≥ 70	59.00	36–104	60.00	38–99	0.2155
Albumin (g/L) > 3.0	3.90	3.3–4.4	3.80	3.27–4.23	0.589
Creatinine (mg/dL) > 2.0	0.90	0.75–1.1	1.00	0.8–1.25	0.735
Platelets (×1000/µL) ≥ 100	155.00	96–219	142.00	95–198	<0.0001
Total bilirubin (mg/dL) > 2.0	1.00	0.7–1.8	1.00	0.7–1.7	0.0514
INR > 1.7	1.20	1.1–1.3	1.17	1.1–1.3	0.0242
Tumor size (cm) > 3.0	2.00	1–3	2.00	1–3	0.6927
SII (×10^9^/L) > 610	452	223–960	409	219–907	0.6943
**HBsAg (+) (*n*, %)**	1034	46.70%	398	17.98%	<0.0001
**HCV antibody (+) (*n*, %)**	535	17.13%	495	15.85%	<0.0001
**MELD Score group (mean, sd)**	8.36	4.83	8.96	5.45	<0.0001
**MELD Score group (*n*, %)**					<0.0001
1	3490	58.43%	1621	27.14%	
2	207	3.47%	171	2.86%	
3	285	4.77%	199	3.33%	
4	0	0.00%	0	0.00%	
**Laboratory data (*n*, %)**					
AFP (ng/mL) ≥ 100	880	13.66%	370	5.74%	0.0018
ALT (U/L) ≥ 70	1124	17.45%	583	9.05%	0.4018
AST (U/L) ≥ 70	1584	24.59%	773	12.00%	0.4870
Albumin (g/L) > 3.0	2532	39.31%	1231	19.11%	0.2108
Creatinine (mg/dL) > 2.0	184	2.86%	126	1.96%	0.0051
Platelets (×1000/µL) ≥ 100	2673	41.50%	1276	19.81%	0.0286
Total bilirubin (mg/dL) > 2.0	823	12.78%	346	5.37%	0.0027
INR > 1.7	124	1.93%	40	0.62%	0.0139
Tumor size (cm) > 3.0	660	10.25%	373	5.79%	0.0389
SII (×10^9^/L) > 610	1097	26.22%	494	11.81%	0.0674

Note: Cases were selected by random 1:2 PSM according to sex, cardiovascular disease, cerebrovascular attack, diabetes mellitus, cirrhosis, hepatitis, hypertension, anti-platelet agent, interferon or nucleic analog used, metformin, NSAID, and statin or fibrate used. All statistical tests were two-tailed and used a type I error rate of 0.05 (p). AFP, alpha-fetoprotein; ALT, alanine aminotransferase; CI, confidence interval, CVD, cardiovascular disease; CVA, cerebrovascular attack; DM, diabetes mellitus; ESRD, end-stage renal disease; HBV, HBsAg positive; HCV, anti-HCV antibody; HR, hazard ratio; INR, International Normalized Ratio; MELD, Model for End-Stage Liver Disease; NSAID, nonsteroidal anti-inflammatory drug; PPI, proton pumping inhibitor; PSM, propensity score matching. Systemic immune-inflammation index (SII) was defined as follows: neutrophil × platelet/lymphocyte.

**Table 2:** Mortality incidence of hepatocellular carcinoma between geriatric and younger patients within a 3-year follow-up (1:2 PSM).

		Case Number	Death	Incidence (%)	Mean Following Year	Total Following Year	Incidence Rate (per 1000 Person-Year)		
Age < 65 years	3 year	4294	2439	56.80%	1.680	7214.15	338.08		
Age 65–75 years	3 year	2147	1269	59.11%	1.634	3508.38	361.70		
		Crude HR	95% CI		*p*-value	Adjusted HR	95% CI		*p*-value
Age < 65 years									
Age 65–75 years	3 year	1.023	0.891	1.175	0.7448	1.108	0.957	1.282	0.1695

Note: Using the Fine–Gray method to account for all-cause mortality as a competing risk of Incident HCC. Cases were selected by random 1:2 PSM according to sex, cardiovascular disease, cerebrovascular attack, diabetes mellitus, cirrhosis, hepatitis, hypertension, anti-platelet agent, interferon or nucleic analog used, metformin, NSAID, and statin or fibrate. CI, confidence interval; HR, hazard ratio; PSM, propensity score matching.

**Table 3:** Analysis of risk factors for mortality in incident patients with hepatocellular carcinoma.

	Univariate Cox Model				Multivariate Cox Model			
	HR	95% CI		*p*-Value	HR	95% CI		*p*-Value
Age 65–75 years	1.0230	0.8910	1.1750	0.7448	1.1080	0.9570	1.2820	0.1695
Sex (Male)	1.2580	1.0450	1.5150	0.0154	1.5380	1.2630	1.8730	<0.0001
HBsAg (+)	0.9710	0.8510	1.1080	0.6618				
HCV antibody (+)	0.9120	0.7920	1.0500	0.2012				
CAD or CVA	1.3580	0.8900	2.0730	0.1559				
Diabetes	0.9870	0.8450	1.1530	0.8690				
Cirrhosis	1.5330	1.3210	1.7800	<0.0001	1.5950	1.3580	1.8730	<0.0001
Hepatitis	0.4230	0.3190	0.5590	<0.0001	0.5570	0.4190	0.7400	<0.0001
Hypertension	0.7900	0.6690	0.9330	0.0054	0.9240	0.7750	1.1030	0.3827
Anti-platelet or aspirin	1.2760	1.0900	1.4930	0.0025	1.0390	0.8750	1.2330	0.6655
Interferons or nucleoside analogue	0.7530	0.6520	0.8690	0.0001	0.7810	0.6700	0.9110	0.0016
Metformin	0.8000	0.6430	0.9960	0.0458	0.9790	0.7800	1.2300	0.8563
NSAID	0.9210	0.8010	1.0590	0.2461				
Statin or Fibrate	0.4640	0.3280	0.6570	<0.0001	0.5710	0.3950	0.8260	0.0029
ALT (U/L) ≥ 70	1.4050	1.2180	1.6220	<0.0001	1.3405	1.1364	1.5798	0.0005
AST (U/L) ≥ 70	2.6890	2.3590	3.0640	<0.0001	1.7870	1.5290	2.0900	<.0001
Albumin (g/L) > 3.0	0.5050	0.4390	0.5810	<0.0001	0.7410	0.6360	0.8640	0.0001
Total bilirubin (mg/dL) > 2.0	2.7600	2.3930	3.1830	<0.0001	1.8520	1.5790	2.1720	<.0001
Creatinine (mg/dL) > 2.0	1.3610	1.0660	1.7360	0.0132	1.0380	0.7300	1.4750	0.8372
Platelets (×1000/µL) ≥ 100	1.1320	0.9790	1.3100	0.0944				
INR > 1.7	2.4380	1.7880	3.3230	<0.0001	2.3280	1.6710	3.2430	<0.0001
SII (×10^9^/L) > 610	2.8760	2.5190	3.2830	<0.0001	1.5180	1.3110	1.7570	<0.0001
AFP (ng/mL) ≥ 100	2.4730	2.1700	2.8190	<0.0001	1.6460	1.4280	1.8980	<0.0001
Tumor size (cm) > 3.0	3.1290	2.6160	3.7420	<0.0001	0.7970	0.6560	0.9680	0.0224
TNM stage								
II	1.9930	1.5960	2.4880	<0.0001	1.7430	1.3920	2.1840	<0.0001
III	7.0920	5.9120	8.5090	<0.0001	5.4860	4.4880	6.7050	<0.0001
IV	13.0480	10.5260	16.1740	<0.0001	9.6060	7.5020	12.2980	<0.0001
MELD Score group								
2	1.6570	1.3040	2.1060	<0.0001	1.3970	1.0920	1.7870	0.0079
3	1.8270	1.5160	2.2010	<0.0001	1.6780	1.2770	2.2040	0.0002

Note: Using the Fine–Gray method to account for all-cause mortality as a competing risk of incident liver-related mortality. PSM variables: sex, age, cardiovascular disease, cerebrovascular attack, diabetes mellitus, cirrhosis, hepatitis, hypertension, anti-platelet agent, interferon or nucleic analog used, metformin, NSAID, and statin or fibrate used. AFP, alpha-fetoprotein; ALT, alanine aminotransferase; CI, confidence interval, CVD, cardiovascular disease; CVA, cerebrovascular attack; DM, diabetes mellitus; ESRD, end-stage renal disease; HBV, HBsAg positive; HCV, anti-HCV antibody; HR, hazard ratio; INR, International Normalized Ratio; MELD, Model for End-Stage Liver Disease; NSAID, nonsteroidal anti-inflammatory drug; PPI, proton pumping inhibitor; PSM, propensity score matching. Systemic immune-inflammation index (SII) was defined as follows: neutrophil × platelet/lymphocyte.

## Data Availability

The datasets used and analyzed in this study are available from the corresponding author upon request.

## Supplementary Materials

The following supporting information can be downloaded at https://www.mdpi.com/article/10.3390/curroncol29060346/s1, Table S1: ICD codes used in this study.
