# Supplementary material for: Outcomes of Geriatric Patients with Hepatocellular Carcinoma
Source: Curr Oncol. 2022 Jun 16;29(6):4332–41. doi: 10.3390/curroncol29060346 (PMC9221899; doi:10.3390/curroncol29060346)
Supplement: Supplementary file 1 [file curroncol-29-00346-s001.zip › curroncol-1738268-supplementary.pdf]

Current Oncology

Article

Outcomes of Geriatric Patients with Hepatocellular Carcinoma

Chern-Horng Lee, Tzung-Hai Yen, and Sen-Yung Hsieh

Supplementary Materials

Table S1. ICD codes used in this study

Group	Co-morbidity disease	ICD-9 code	ICD-10 code
1	Cardiovascular disease (CVD)		
	Angina pectoris,	4139	I20
	Acute myocardial infarction	410, 4109	I21.9
	Athersclerosis	4409	I25.1
	Coronary arterial disease (CAD)	4140	I25.10
	Old myocardial infarction	412	I25.2
	Other acute ischemic heart disease		I24
	Ischemia heart disease (IHD)	4149	I25
	Certain current complications following ST elevation (STEMI) and non-ST elevation (NSTEMI) myocardial infarction (within the 28 days period)		I22, I23
2	Cerebral vascular accident (CVA)		
	Transient ischemia attack (TIA)	435, 4359	G45.9, G45
	Cerebral Infarction	4349	I63
	Stroke	433–436, 4370	G46
	Squeal of Stroke	438.9	I69.3
3	Diabetes mellitus (DM)	2500, 25001, 25050, 25060, 3572, 25080, 250, 25070, 2507, 7854	E11, E10
4	Cirrhosis	5712, 5715, 5716	K702, K7030, K7031, K74, K 74.60, K70.3
5	Biliary cirrhosis		K74.5
6	HBsAg	7030	B16
7	Anti-HCV antibody	7051	B18
8	Hepatitis	57140, 5710, 5711	K73 K70.1 B17
9	Hepatocellular carcinoma	1550	C22.0
10	Hypertension	401-405	I10-I15
